# A study of auxiliary factors for judges in sentencing dangerous driving offenses – the mediating role of favorable and unfavorable factors

**DOI:** 10.3389/fpsyg.2023.1202518

**Published:** 2023-09-19

**Authors:** Chengjie Liu, Ziyu Miao, Ting Wang

**Affiliations:** ^1^School of Humanities and Social Sciences, University of Science and Technology Beijing, Beijing, China; ^2^The Dickson Poon School of Law, King’s College London, London, United Kingdom; ^3^School of Law, New York University, New York, NY, United States

**Keywords:** dangerous driving offenses, sentencing, auxiliary factors, mediation, psychological tendencies

## Abstract

This paper investigates the ancillary factors judges consider when sentencing dangerous driving offenses. These factors are divided into favorable (i.e., “Confess” [*CF*], and “Actively compensate and obtain forgiveness from victims” [AC]) and unfavorable categories (i.e., “Mainly or solely responsible for the accident” [MSR], and “Post-accident behavior” [BAA]). Results indicate that both types of factors mediate the relationship between “Blood Alcohol Concentration” (BAC) and the sentencing outcome: favorable factors have a significant negative correlation with sentences, while unfavorable ones show a positive correlation. The mediation effect ratios are 13.8% and 19.1% respectively, with no significant differences between their impacts on judges’ sentencing decisions.

## 1. Introduction

Sentencing means the formal judgement of whether to impose a sentence on the offender, what type of sentence to impose, and how heavy the sentence to impose, based on the identification of the facts of the crime and the nature of the crime in accordance with the applicable laws and regulations. All along, due to the lack of empirical research in Chinese theoretical and practical fields, it is concluded that the sentencing of Chinese judicial courts is imbalanced. By a case-by-case basis method, random assignment method, sentencing experiment method, and data comparison method, Cai (2013) concluded after analysis that sentencing imbalance does exist in Chinese judicial practice (p. 82). The imbalance in sentencing shows to some extent the severity of “different sentences for the same case,” thus it is necessary to study the factors that influence judges’ decisions on imposing a sentence.

### 1.1. Auxiliary factors for the judge’s sentencing

In addition to the main factors, mainly the constitutive elements involved in the case, a variety of factors such as the judge’s character traits, suspect’s characteristics, and post-crime behavior need to be considered when the judge imposes a sentence (Li and Ma, 2014, p. 197; Jiang, 2015, p. 159; Zhang, 2015, p. 2). Li et al. (2015) concluded that judges’ emotional preferences on the case significantly influence their sentencing (p. 200). They tend to impose a shorter sentence with sympathy but a longer one with disgust and anger (Wang, 2004, p. 11; Li, 2013, p. 107; Li and Ma, 2014, p. 199).

Neither gender nor time in practice significantly arises the judges’ emotion, no matter whether relevant or irrelevant to the case (Zhang, 2015, p. 4). However, Lao (2022) does not agree with that view and argued that the offender’s gender does influence the sentence judgement of the judge case by case: judges tend to “favor men over women,” that is imposing longer sentences on men in the sentencing of intentional homicide offenses, while displaying the opposite bias in the sentencing of fraud and drug trafficking offenses. Only in cases of robbery, the sentencing outcomes achieve gender equality, without a unified trend of favoring males over females as advocated by traditional views (p. 103).

Li (2008) believed that in criminal trials, besides the convicted circumstances and given circumstances, external factors such as the supervision of the superior, opinions and attitudes of the public may also influence judges’ sentencing under the specific circumstances (p. 130). Zhou (2016) argued that the education level of the judges may also affect the sentence. There are a great number of judges in Chinese judicial system who have not any legal training, and many of them are military veteran becoming a judge whose educational levels varied from person to person, and some of them even have not obtained professional qualifications thus resulting of a great disparity of their judgements of a sentence (p. 107).

Sun (2005) argued that the sentencing mechanisms itself also influence judges’ sentencing: some cases were in accordance with the statutory law, some follow the precedents, some refer to the guidelines of sentencing, some adopt prosecutors’ recommendations on the sentence, while others depend on judges’ discretion (p. 54). For example, Teske and Albrecht (1992) pointed out that according to the German Code of Criminal Procedure, defendants, prosecutors, and courts can enter into plea bargains, indicating that plea bargains can also have a large impact on judges’ sentencing (p. 85). According to Minoru (2000), the underlying facts related to sentencing can be divided into two categories: circumstances that are part of the facts of the crime, including the motive, method, result, and social impact of the crime, and circumstances that are not part of the facts of the crime, including the age, character, experience, and situation of the prisoner, and his or her performance after the crime (p. 29).

### 1.2. The position of auxiliary factors in the sentence of dangerous driving offense

During the first two years following the promulgation of the Amendment VIII of the Criminal Law of the People’s Republic of China, the legal framework was relatively incomplete, and the number of cases involving dangerous driving offenses was not substantial. As a result, judicial personnel took the sentencing for dangerous driving offenses cautiously. On December 18, 2013, the Supreme Court, the Supreme Prosecutor, and the Ministry of Public Security jointly issued Opinions on Several Issues Concerning the Application of Law in Handling Criminal Cases of Drunken Driving of Motor Vehicles, which regulated the application of dangerous driving offenses. Under the Opinions on Several Issues Concerning the Application of Law in Handling Criminal Cases of Drunken Driving of Motor Vehicles: whoever drives a motor vehicle on the road with his or her blood alcohol content reaching 80 mg per 100 mL or more shall be deemed to be driving a motor vehicle while intoxicated and be punished for the crime of dangerous driving in pursuant to Paragraph 1 of Article 133(A) of the Criminal Law.

It is evident that under the Criminal Laws of the People’s Republic of China, when judging the dangerous driving offense, the determination of drunk driving takes the driver’s BAC as the only crime criterion (not equal to sentencing criteria), which constitutes a typical abstract dangerous offense. This differs from the judgements of many overseas jurisdictions. For example, under the governing of German laws, although BAC is a criterion to be considered, the driver’s capability to drive at the time of the conduct determines whether the driver is guilty of dangerous driving. Due to dangerous driving being an abstract crime, where the commission of a corresponding type of dangerous behavior constitutes the offense, compared to other criminal cases, the facts and evidence in dangerous driving cases are easier to identify. The proportion of voluntary pleas from the parties involved is higher, and the difficulty in handling these cases is lower, resulting in shorter case durations. These factors have led to a significant increase in the number of dangerous driving offenses since 2013. Dangerous driving crimes, especially those involving drunk driving, have become a common and frequent type of case in grassroots criminal justice practice (Tian, 2023, p. 178). Standardizing and refining the sentencing guidelines for dangerous driving offenses can facilitate the diversion of cases based on their complexity, thereby enhancing judicial efficiency.

In the judicial practice of dangerous driving cases, there still exist issues such as inaccurate sentencing recommendations, incomplete extraction of sentencing factors, and non-standardized evaluation of sentencing circumstances. These problems have led to imbalanced sentencing, inconsistent criteria for probation application, and varying degrees of excessive or lenient sentencing worldwide (Wu and Li, 2017, p. 139). The analysis of dangerous driving judgements reveals a pattern of heavier convictions and lighter punishment by the courts (Kury et al., 2009, p. 64; Su and Wang, 2018, p. 141).

In practice, the sentencing of dangerous driving cases is primarily influenced by four aspects: firstly, the factual circumstances of the actual danger, including blood alcohol content, time and location of the offense, type of vehicle involved, presence or absence of vehicle registration plates, the extent of damage caused by the accident, and the nature of the violation. Yin and Wang (2022) concluded that there is a strong linear correlation between the blood alcohol content and the length of detention sentenced to the defendant (p. 189). By analyzing 4,782 sentences nationwide, Zhang and Li (2014) found that the highest average prison term for crimes committed in the afternoon is 2.114 months, while the lowest average prison term for crimes committed in the early morning is 1.785 months. Notably, the prison terms during the period from dawn until noon are significantly lower than those in the afternoon, reflecting the varying levels of social danger caused by dangerous driving during each time period (p. 104). Wu and Wang (2019) argued that the location of the accident on a roadway is also an important factor in sentencing. For instance, dangerous driving incidents occurring in busy areas are considered more severe than those occurring in suburban areas, and this should be reflected in the sentencing process (p. 82). In addition, Zhang and Li (2014) believed that the occurrence of a single-vehicle accident should also be considered a mitigating factor in sentencing. From the perspective of accident damage, a single-vehicle accident only causes harm to the rights of the intoxicated driver themselves, either in terms of personal injury or property damage. Unlike general accidents that cause harm to the personal and property rights of third parties, the sentencing for single-vehicle accidents should be lenient rather than severe (p. 108).

The second is that the sentencing factors related to personal danger include criminal records, admission of guilt and remorse, and whether there are any circumstances of voluntary surrender. Tian (2023) through case analysis, suggests that for cases involving dangerous driving under the influence, for first-time or occasional offenders, their subjective malevolence is relatively low, and the level of danger to society is not significant. Therefore, when considering sentencing, greater emphasis should be placed on applying for probation and exemption from punishment in accordance with the law (p. 191). According to Tian (2023), the circumstances of voluntary surrender in cases of dangerous driving under the influence should be distinguished based on different situations. The confession made before the alcohol test constitutes voluntary surrender, while the confession made after the alcohol test only constitutes a truthful confession (p. 192). Thirdly, the mitigating factor of restorative justice sentencing is considered by the Prosecution Office of Leshan, Sichuan Province. If the defendant takes the initiative to restore the social relationships damaged by their dangerous driving behavior and obtains forgiveness from the victim, the judicial trial should positively evaluate this situation during sentencing. Fourthly, the sentencing factor of criminal execution primarily depends on the defendant’s attitude towards admitting guilt and accepting punishment. Defendants who voluntarily pay fines are granted certain sentencing benefits but should not be excessively favored.

From the standardized coefficients of the national regression model, it can be observed that there are numerous factors influencing the sentencing of dangerous driving offenses in practice (Albrecht, 2013, p. 217; Yin and Wang, 2022, p. 197). Wen (2016) also concluded that the relative importance of factors affecting imprisonment sentencing is as follows: “blood alcohol concentration,” region, attitude towards compensation, other aggravating or mitigating circumstances, legitimate license plate, vehicle type, voluntary surrender, and admission of guilt (collectively as “other circumstances”) (p. 170).

The ambiguity of sentencing factors and the discretionary application of these factors make it difficult to ensure consistency in the punishment for similar cases and maintain a “same crime, same punishment” principle. In this context, if it is possible to quantify the sentencing factors of this offense in a statistical form, as a reference for judges, it will enhance the standardization of sentencing and meet the requirements of proportionate punishment.

Existing study has shown a strong linear correlation between BAC and the sentence length, suggesting a direct connection between the two indicators and the sentence length. However, few studies have examined the correlation between BAC and other circumstances (Zhang and Li, 2014, p. 105; Wen, 2016, p. 172). This study proposes that BAC may influence other circumstances of the defendant, thereby aggravating or mitigating their punishment. In other words, these other circumstances occur under the influence of BAC and play a role in augmenting or reducing the defendant’s sentencing. This role played by other circumstances is referred to as “mediation” This paper will employ statistical methods to analyze the mediating effect of other circumstances and demonstrate the impact of these factors on sentencing, thereby offering more certainty in the determination of sentencing for criminal offenses.

### 1.3. Research hypothesis

In this paper, we have extracted 2,896 dangerous driving cases from the Chinese Judicial Documents Online, selected BAC as the basic variable, extracted the auxiliary factors which affect judges’ sentencing, and divided them into two categories based on whether the driver who was charged with dangerous driving could take advantages and disadvantages from these factors. The favorable factors include “Confess” (*CF*) and “Actively compensate and obtain forgiveness from victims” (AC). The unfavorable factors include “Mainly or solely responsible for the accident” (MSR) and “Behavior on the spot after an accident” (BAA). To explore the impacts of BAC, favorable factors, and unfavorable factors on the sentence length imposed by judges, we propose the following research hypothesis based on the previous research findings (Zhang and Li, 2014, p. 105; Wen, 2016, p. 172).

We assume that ancillary factors (i.e., the favorable and unfavorable factors mentioned above) will have an impact on sentence length. Actually, the sentence length is also indirectly affected by BAC, the statutory element (i.e., the “mediating effect” mentioned above). Therefore, we may first discuss whether the other factors that have an impact on sentence length:

*Hypothesis 1:* Favorable factors show a negative correlation with the length of the judge’s sentencing sentence.

*Hypothesis 2:* Unfavorable factors show a positive correlation to the length of the judge’s sentencing sentence.

Moreover, there are existing studies focus more on the impact of BAC and regard it as the sole factor influencing the judgements of sentence. Unfortunately, the correlation between BAC and the favorable and unfavorable factors considered by judges in sentencing has not been clarified. For the causal relationship between BAC and favorable and unfavorable factors, there are merely a few empirical studies using favorable and unfavorable factors as mediators of the impact of BAC (Zhang and Li, 2014, p. 105; Wen, 2016, p. 172). To understand the relationship between the above factors, analyze the impact of BAC on sentence length, we propose the following hypothesis by treating favorable and unfavorable factors as mediating variables.

*Hypothesis 3:* BAC has a positive correlation with unfavorable factors.

*Hypothesis 4:* BAC has a negative correlation with favorable factors.

*Hypothesis 5:* Unfavorable factors mediate the relationship between BAC and length of sentencing.

*Hypothesis 6:* Favorable factors mediate the relationship between BAC and the length of sentencing.

## 2. Materials and metals

### 2.1. Participants

This research is approved by the Research Ethics Committee of the School of Humanities and Social Sciences, University of Science and Technology Beijing. This study extracted 2,896 dangerous driving cases from the Chinese Judicial Documents website, which is part of the Supreme People’s Court of the People’s Republic of China, and all data from the website are authorized for free use by the researcher.

### 2.2. Procedure

The 2,896 cases of dangerous driving convictions in the paper were all downloaded free of charge from the Chinese Judicial Documents website. Then the researcher unified the verdict cases, and extracted and collected *CF*, AC, MSR, and BAA into a database.

### 2.3. Measures

#### 2.3.1. Blood alcohol concentration

We extracted BAC from the judgements and assigned a value to the code, assigned BAC less than 20 mg/100 mL to 1, BAC of 21–80 mg/100 mL to 2, and BAC of 21–80 mg/100 mL to 2. 100 mL as 1, BAC of 21–80 mg/100 mL as 2, BAC of 81–150 mg/100 mL was assigned as 3, and BAC of more than 150 mg/100 mL was assigned as 4.

#### 2.3.2. Favorable factors

We extracted *CF* and AC from the judgements as favorable factors considered by judges to impose a sentence, and carried out coding assignments. The value of “Yes” was assigned as 1, and the value of “No” was assigned as 0.

#### 2.3.3. Unfavorable factors

We extracted MSR and BAA from the judgements as unfavorable factors considered by judges to impose a sentence, and carried out coding assignments. As for MSR, we assigned the value of “Yes” to 1, and assigned the value of “No” to 0. Then, we also assigned a value of 0 for “fit check,” 1 for “reject or prevent check,” and 2 for “escape” in BAA.

#### 2.3.4. Sentence length

The sentence length extracted from the sentencing case were regarded as a true value record. The final way of encoding the formation data files is shown in Table 1.

**Table 1:** Data assignment table.

Factor		Assignment
Unfavorable factors	“Mainly or solely responsible for the accident”	0- No;
		1- Yes
	“Behavior on the spot after an accident”	0- Cooperate with inspection;
		1- Refuse or obstruct the inspection;
		2- Escape
Favorable factors	“Confess”	0- No;
		1- Yes
	“Actively compensate and obtain forgiveness from victims”	0- No;
		1- Yes
“Blood alcohol concentration”		1- Not more than 20mg/100ml;
		2- 21–80mg/100ml;
		3- 81–150mg/100ml;
		4- Greater than 150mg/100ml

### 2.4. Data analysis

This study first discusses the relationship between variables using Spearman correlation analysis and then analyzes the relationship between BAC, favorable and unfavorable factors by using structural equation modeling based on the mediation analysis process proposed by Wen and Ye (2014, p. 732). The bootstrap method will be used to test the significance of the mediating role of favorable and unfavorable factors in BAC and sentencing length, and robust standard errors and confidence intervals are obtained for the parameter estimates. If the confidence interval does not include zero, the statistical results are significant (Erceg and Mirosevich, 2008, p. 594).

## 3. Results

### 3.1. Common method deviation test

To reduce the common method deviations caused by self-reported questionnaires, we emphasized the authenticity of the answers during the data collection process; the scale and the order of the questions are randomly set for program control. We will use Harman’s single factor test to test the effect of program control (Podsakoff et al., 2003, p. 889), while exploratory factor analysis is conducted on three variables. It is found that after rotation, the characteristic roots of 8 factors are greater than 1, and the explanatory rate of the first factor is 17.23% (far less than the critical value of 40%), which indicates that the degree of variation in the common method used in this study is within the acceptable range, meaning the potential data bias of this study is in the normal range.

### 3.2. Descriptive and bivariate analyzes

The mean values, standard deviations, and intercorrelations of the variables are shown in Table 2. In Table 2, the numbers 1 through 6 in the first row correspond one-to-one with the first column, indicating the direct relationship between those elements. If the calculation meets the requirements of “*p < 0.05 or **p < 0.001,” it can be concluded that there is a correlation between the two elements. As shown in Table 2, sentence length in the sixth row is significantly correlated with all elements, indicating that BAC and unfavorable factors are positively correlated with the sentence length, while favorable factors are negatively correlated with the sentence length. On this basis, it can be concluded that hypothesis 1 (*Favorable factors show a negative correlation with the length of the judge’s sentencing sentence*) and 2 (*Unfavorable factors show a positive correlation to the length of the judge’s sentencing sentence*) are valid.

**Table 2:** Means, standard deviations, and intercorrelations for variables.

	*M*	SD	1	2	3	4	5	6
1. MSR	0.19	0.394	1.000					
2. BAA	0.05	0.289	0.121^**^	1.000				
3. AC	0.39	0.488	−0.027	0.003	1.000			
4. CF	0.36	0.480	0.004	−0.003	0.041^*^	1.000		
5. Blood alcohol concentration	3.38	0.660	0.134^**^	0.061^**^	−0.074^**^	0.004	1.000	
6. Sentence length	2.02	1.072	0.211^**^	0.090^**^	−0.037^*^	−0.070^**^	0.381^**^	1.000

*N* = 2896, *p<0.05, **p<0.001. “*p*” stands for the significance of the test and “*” stands for the level of significance. The smaller the value of “*p*” and the greater the amount of “*”, the stronger the relationship between the two variables. “*M*”, means; “SD”, standard deviations. The numbers 1 through 6 in the first row correspond to the numbers 1 through 6 in the first column. For example, 1 = 1. MSR, 2 = 2. BAA, …, 6 = 6. Sentence length.

### 3.3. Intermediary model checking

To test hypothesis 1 and 2, the structural equation model is used to investigate the impact of BAC, favorable factors, and unfavorable factors on sentencing length in Table 2.

**Figure 1:**
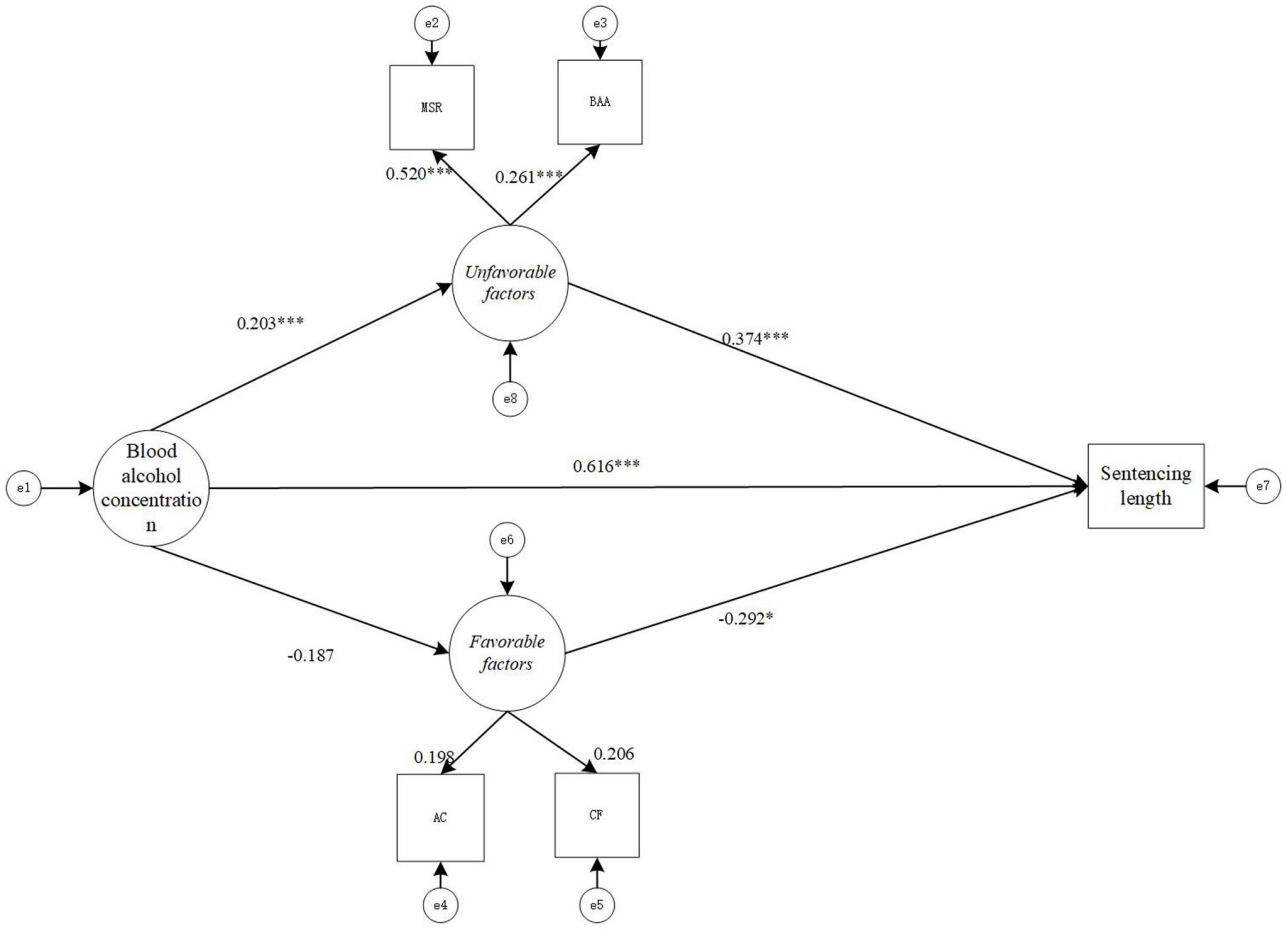
Structural equation intermediary relationship model diagram. *p<0.05, **p<0.01, ***p<0.001. “MSR”, mainly or solely responsible for the accident; “HLP”, have been legally punished for driving a motor vehicle while drinking; “DL”, driving without a license; “BAA”, behavior on the spot after an accident; “VPG”, voluntarily plead guilty in court; “CF”, confess; “AC”, actively compensate and obtain forgiveness from victims; “e”, error (residual). The “e” here reflects the randomness of the data, making the calculations more credible.

In Table 2, both favorable factors, and unfavorable factors are taken as direct influences. In the next argumentation, this study will utilize the intermediary analysis process proposed by Wen and Ye (2014, p. 732) once again to build a statistical model, by using the favorable factors, and unfavorable factors as indirect influencing factors (i.e., mediating factors) to perform the arithmetic. The final constructed model and the calculation results can be seen in Figure 1.

Before the mediating effect analysis, the maximum likelihood estimation method has been used to test the hypothesis model in Figure 1. The fitting index indicators of the structural equation model are: χ^2 (6) =15.741, CFI = 0.979, TLI = 0.948, SRMR = 0.013, RMESA = 0.024, the 90% confidence interval of RMSEA is [0.010, 0.038], and the results show that the model fits well.

### 3.4. Mediating effect analysis

From the path diagram of the relationship between BAC, favorable factors, unfavorable factors, and sentence length (Figure 1), it can be seen that BAC positively predicts unfavorable factors (γ = 0.205, *p* < 0.001), and BAC negatively predicts favorable factors (γ = −0.298, *p* < 0.001), while unfavorable factors (γ = 0.375, *p* < 0.001) positively predict sentencing length, favorable factors (γ = −0.326, *p* < 0.05) negatively predict sentencing length. Thus hypothesis 3 (*BAC has a positive correlation with unfavorable factors*) and 4 (*BAC has a negative correlation with favorable factors*) are valid.

The direct impact of BAC on sentencing length is significant (γ = 0.092, *p* = 0.082). Based on the mediation model in Figure 1, the non-parametric percentile Bootstrap method (with a sampling number of 5,000 and a confidence interval of 95%) is used to further test the significance of the mediating effect. The results show that unfavorable factors have a Partial mediation effect between BAC and sentencing length (mediating effect = 0.123, SE = 0.037, *p* < 0.005, 95% CI = [0.064,0.205]), while the data of favorable factors is (mediation effect = 0.089, SE = 0.113, *p* = 0.434, 95% CI = [0.002, 0.713]).

It can be concluded that both favorable factors and unfavorable factors partially have a mediating effect on BAC. The mediating effect of unfavorable factors is 0.123/(0.123 + 0.089 + 0.431) = 0.191 (19.1%), while the mediating effect of favorable factors is 0.089/(0.123 + 0.089 + 0.431) = 0.138 (13.8%).

The Sobel test can also be used to test the significance of the mediation effect (Sobel, 1982, p. 292). The calculation results are shown in Table 3.

**Table 3:** Sobel test results.

		*z*	S.E.	*p*
a_1_	0.203	4.14269242	0.01832673	0
S_a1_	0.374			
b_1_	5.497			
S_b1_	0.878			

a_1_ is the regression coefficient of unfavorable factors and Blood alcohol concentration, S_a1_ is the corresponding S.E., b_1_ is the regression coefficient of unfavorable factors and sentence length, and S_b1_ is the corresponding S.E.

It can be seen from Table 3 that the mediating effect of favorable factors between BAC and sentence length is significant (*z* = 3.33, *p* < 0.05), and it can also be found on unfavorable factors (*z* = 3.71, *p* < 0.05). Therefore, hypothesis 5 (*Unfavorable factors mediate the relationship between BAC and length of sentencing*) and 6 (*Favorable factors mediate the relationship between BAC and the length of sentencing*) are valid.

## 4. Discussion

According to the applicable Chinese criminal laws, BAC in the range of 20–80 mg/100 mL is the crime conduct of drinking and driving, and BAC greater than 80 mg/100 mL is the crime of drunk driving, and the sentence imposed for drunk driving will be longer. So, the judge will first make a judgement based on the driver’s BAC and then take into consideration of favorable and unfavorable factors to make a comprehensive and final judgement.

According to the study results, BAC, unfavorable factors and favorable factors all showed a significant correlation with the sentence length, in which the data is (γ = 0.381, *p* < 0.001), (γ = 0.374, *p* < 0.001) and (γ = −0.292, *p* < 0.05) respectively.

### 4.1. The impact of unfavorable factors on the sentence length

There is a significant positive correlation between the unfavorable factors and the sentence length. The relationship between the unfavorable factors MSR and the sentence length is found to be significantly positive (γ = 0.211, *p* < 0.001), as well as BAA (γ = 0.090, *p* < 0.001). It indicates that the primary responsibility the drunk driver shall take for the accident he or she caused, followed by the driver’s post-accident behavior, is the focus of the judge’s sentencing, and whether the driver has been punished before is irrelevant. Therefore, the determination of accident responsibility becomes a key unfavorable factor in sentencing.

In addition, according to the applicable laws and regulations, if the driver is not qualified, such as driving without a license and flees from the accident scene, he or she shall take full or primarily responsible for the accident. In the data analysis, it can be found that MSR shows a significant positive correlation with BAA, which is (γ = 0.122, *p* < 0.001).

### 4.2. The influence of favorable factors on the sentence length

There is a significant negative correlation between favorable factors and sentence length. According to the Table 2, it is found that AC among the favorable factors has a significant negative correlation with sentence length (γ = −0.037, *p* < 0.001), followed by *CF* (γ = −0.070, *p* < 0.001).

It indicates that judges focus on the driver’s *CF* situation when sentencing, followed by the AC, and it also proves that judges will look into the primary responsibility of the accident when sentencing, and adopt the guideline of “leniency for confession and severity for resistance.” Judges will also take into consideration of driver’s compensatory behavior, although it is not the primary basis for the judges to mitigate the sentence.

### 4.3. The mediating role of favorable and unfavorable factors on the sentence length

The favorable factor plays a partial mediating role between BAC and sentence length, and the proportion of the mediating effect is 13.8%. From the data analysis, we also find that AC among the favorable factors shows a significant negative correlation with BAC (γ = 0.074, *p* < 0.001). This indicates that when the driver does not drink much alcohol, i.e., the BAC is not high, the driver is likely to take less responsibility for the accident and the sentence will be relatively shortened; if the driver is inclined to compensate the victim and plead guilty, the judge would like to impose a shorter sentence.

The unfavorable factors partially mediate the relationship between BAC and sentence length, with a mediating effect of 19.1%. From the data analysis, it can also be found that the unfavorable factor MSR and BAC shows a significant positive correlation (γ = 0.136, *p* < 0.001), and the on-the-spot behavior after traffic accidents caused by drunk driving shows a significant positive correlation with BAC (γ = 0.061, *p* < 0.001). This indicates that the more alcohol the drivers drink, they are more likely to have unclear minds, delayed and impulsive behavior thus resulting in main or full responsibility to take in the accident, and even resisting law enforcement and escaping from the accident scene, which definitely would lead to a longer sentence.

The mediating effect of the unfavorable factor outweighs the favorable factor but fails in conducting a bootstrap test (diff = 0.033, SE = 0.151, *p* = 0.572 > 0.1, 95% CI = [−0.681, 0.097]), indicating that there is not a large disparity between the impact of two factors on the judges’ sentencing.

### 4.4. Going forward

In China, Dangerous Driving Offense, as a kind of abstract dangerous crime, exhibits variations in sentencing, even among similar offenses. Based on the results of the analysis above, this paper would like to provide solutions to this issue from the following perspectives.

On the one hand, the analysis in this paper indicates that there is a strong correlation between BAC and sentence length. It means that BAC is the most important basis for judges to make judgements. When rendering a judgement, the judges are expected to follow the precedents of the offense first and then impose the sentence based on BAC. At the same time, according to the applicable law and relevant judicial interpretations, relevant elements of the crime (such as “driving without a license,” etc.) shall also be considered to shorten or extend the sentence length.

On the other hand, BAC and other relevant criminal factors do not typically result in significant variations in sentencing within collective cases. The primary cause of this issue lies in certain secondary factors, which have been analyzed in the paper. Previously, many judgements excessively emphasized these secondary factors to the extent that they even determined the sentencing. However, through statistical analysis, it can be observed that these secondary factors serve as mediators. In other words, BAC has a certain influence on these secondary factors, which ultimately determine the sentence length. On the surface, the secondary factors may increase or decrease the sentence length, however, fundamentally, it is a result of the indirect impact exerted by BAC.

All in all, in China, to avoid “different sentences for the same case,” judges shall consider BAC and the crime factors explicitly stipulated by the law as the sole determining criteria. Given that the mediating roles of secondary favorable and unfavorable factors (i.e., the four elements analyzed in this paper) are 13.8 and 19.1% respectively, and that there is no significant disparity between favorable and unfavorable factors in sentencing, this paper proposes that the influence of the secondary factors on the final judgement of sentence should not be less than 10%, but not more than 20%.

### 4.5. Limitations

The paper mainly discusses the impact of unfavorable and favorable factors on the judges’ judgement of sentence length and the mediating effect of favorable and unfavorable factors between BAC and the sentencing period. However, the interaction between favorable and unfavorable factors is not addressed herein, and the impact of the intersection of the two factors on the judge’s sentence would be further analyzed in the future.

## 5. Conclusion

This paper analyzes the impacts of unfavorable and favorable factors on judges’ sentence judgement and the mediating effect of favorable and unfavorable factors between BAC and sentence length.

A significant negative correlation is found between favorable factors and sentence length, and a significant positive correlation is found between unfavorable factors and sentence length. The favorable factors and the unfavorable factors are both partially mediated between BAC and sentence length. While the mediating effect ratio of the former is 13.8%, the data of the latter is 19.1%. Meanwhile, there is no significant difference between favorable and unfavorable factors in sentencing.

On this basis, we hold the view that judges shall value the impact of BCA and related offense factors on sentence length, and further clarify their impact extent at sentencing. Moreover, the judges shall minimize the impact of favorable and unfavorable factors in the final judgement. Thus quantitative and standardized indicators for sentencing would become more important factor and show a great proportion in the judgements of Dangerous Driving Offenses, and conversely, abstract and auxiliary one’s effects will be minimized to avoid “different sentences for the same case.”

## Data availability statement

The original contributions presented in the study are included in the article/Supplementary material, further inquiries can be directed to the corresponding author.

## Ethics statement

Studies involving human participants were reviewed and approved by the University of Science and Technology Beijing Research Ethics Committee, and express consent from the patients/participants or patients/participants’ legal guardians was not required in this study in accordance with the national legislations and the institutional requirements.

## Author contributions

CL contributed to the conception, design of the study, data collection, and performed the statistical analysis. TW and ZM contributed to preparation and revision of paper. All authors contributed to the article and approved the submitted version.

## Funding

This research has received support from the National Natural Science Foundation of China (L2124028).

## Conflict of interest

The authors declare that the research was conducted in the absence of any commercial or financial relationships that could be construed as a potential conflict of interest.

## Publisher’s note

All claims expressed in this article are solely those of the authors and do not necessarily represent those of their affiliated organizations, or those of the publisher, the editors and the reviewers. Any product that may be evaluated in this article, or claim that may be made by its manufacturer, is not guaranteed or endorsed by the publisher.

## Supplementary material

The Supplementary material for this article can be found online at: https://www.frontiersin.org/articles/10.3389/fpsyg.2023.1202518/full#supplementary-material
